# On Gait Analysis Estimation Errors Using Force Sensors on a Smart Rollator

**DOI:** 10.3390/s16111896

**Published:** 2016-11-10

**Authors:** Joaquin Ballesteros, Cristina Urdiales, Antonio B. Martinez, Jaap H. van Dieën

**Affiliations:** 1 Department of Electronic Technology, University of Malaga, 29071 Malaga, Spain; acurdiales@uma.es; 2 Department of Automatic Control, Polytechnic University of Catalonia, 08034 Barcelona, Spain; antonio.b.martinez@upc.edu; 3 MOVE Research Institute Amsterdam, Department of Human Movement Sciences, Vrije Universiteit Amsterdam, 1081 BT Amsterdam, The Netherlands; j.van.dieen@vu.nl

**Keywords:** gait characterization, smart rollator, assistive devices, disability profiling

## Abstract

Gait analysis can provide valuable information on a person’s condition and rehabilitation progress. Gait is typically captured using external equipment and/or wearable sensors. These tests are largely constrained to specific controlled environments. In addition, gait analysis often requires experts for calibration, operation and/or to place sensors on volunteers. Alternatively, mobility support devices like rollators can be equipped with onboard sensors to monitor gait parameters, while users perform their Activities of Daily Living. Gait analysis in rollators may use odometry and force sensors in the handlebars. However, force based estimation of gait parameters is less accurate than traditional methods, especially when rollators are not properly used. This paper presents an evaluation of force based gait analysis using a smart rollator on different groups of users to determine when this methodology is applicable. In a second stage, the rollator is used in combination with two lab-based gait analysis systems to assess the rollator estimation error. Our results show that: (i) there is an inverse relation between the variance in the force difference between handlebars and support on the handlebars—related to the user condition—and the estimation error; and (ii) this error is lower than 10% when the variation in the force difference is above 7 N. This lower limit was exceeded by the 95.83% of our challenged volunteers. In conclusion, rollators are useful for gait characterization as long as users really need the device for ambulation.

## 1. Introduction

Currently, a high percent of population presents some form of disability. For example, in Europe, 18% of the population over 16 years old report moderate disabilities [1]. Some of these disabilities affect mobility, which is of key importance for being independent. Mobility analysis, and particularly gait analysis, plays an important role in rehabilitation [2], early detection of degenerative processes [3] and diagnostics [4].

Gait analysis is usually performed in a controlled, instrumented area. Data capture can rely on sensors in the walking surface, such as force plates in a treadmill [5] or pressure sensors in a walkway [6]. Additionally or alternatively, it can rely on an optical motion capture system. A variety of optical sensors are in use, including stereovideography [7], active-marker systems [8], infrared thermography [9], Time-of-Flight Systems (ToF) [10] and structured light patterns [11]. All of these solutions constrain data capture to sensorized areas, and they require an expert to setup and control the system. These measurement conditions may be distressing for individuals to be assessed, especially when walking velocities are externally imposed. In addition, monitoring is limited in duration and limited in terms of walking trajectory and environment.

Gait analysis has also been performed using wearable sensors. Users may carry a number of sensors on various part of their bodies, such as pressure sensors under the feet [12], inertial sensors attached to shanks and thighs [13] or on the trunk [14]. Consistency of gait characteristics are determined from acceleration data collected at different trunk locations [15] or electromyogram (EMG) electrodes attached over muscles of interest [16]. These solutions are not always comfortable to users because they must carry sensors attached to their bodies, so they are usually worn for limited time periods.

To improve usability and to perform gait analysis in daily life, sensors could be attached to a device that people use on a daily basis. One solution is to use inertial sensors in a smartphone [17], but assistive devices like walkers, rollators, canes, etc. may support a larger number of relevant sensors. Specifically, force sensors in walkers and rollators provide relevant gait information, as users bear more weight on one handlebar or the other depending on the foot they are using for support at the moment. Hence, such sensors indirectly provide information about heel strike, which can be used to obtain several gait parameters. Rollators are frequently used in rehabilitation by people who require a larger base of support and moderate weight bearing [18]. If these devices are equipped with force sensors, they can provide a continuous data flow for gait analysis.

Instrumented or smart rollators have already been used for gait analysis using optical information, where the users’ feet were tracked by Microsoft Kinect sensors (Microsoft, Redmond, WA, USA) [19] and Time of Flight (ToF) cameras [20] attached to the frame. The main drawbacks of these vision based solutions are that: (i) they are limited to controlled illumination areas; (ii) they require some skill to set up and operate; and (iii) they return a high bit stream. In addition, ToF cameras are quite expensive. Force based gait analysis can be performed with on board force sensors fully integrated in the rollator frame. These sensors are cheap, reliable and invisible to the user, and they return a low bit stream that can be easily processed on the fly. In [21,22], for example, gait parameters were obtained from force sensors on the rollator handlebars. Similar studies used 3D accelerometers/gyroscopes attached to the frame to obtain gait parameters [23], but this approach has only been tested on three-wheeled rollators, where gait-induced frame rotation is more sensitive to force balance.

Rollator based gait analysis approaches typically present two major weaknesses. First, solutions are usually tested using healthy volunteers for simplicity. Unfortunately, healthy people do not usually present the same behavior as challenged users when they handle assistive devices. Much work has focused on comparing healthy gait and the specific gait of challenged volunteers due to e.g., Parkinson’s [24], stroke [25] or ataxia [26]. One of the major differences between healthy volunteers and the challenged volunteers is the amount of weight supported by the devices. Consequently, tests using healthy people may not be valid, especially not in the case of force based gait analysis. A second weakness is that force based gait analysis is bound to be less accurate than more direct estimates derived from floor sensors, optical systems or wearable sensors.

Nevertheless, force based gait estimation is quite appealing because: (i) it is not constrained to any specific environment; (ii) it does not require expert intervention; and (iii) users do not need to wear sensors themselves. In addition, data can be processed on the fly to provide continuous information on gait, anytime, anywhere. We already proposed a method for gait analysis using a rollator in [22]. The present study had two objectives: (i) to define the target population for the proposed methodology; and (ii) to determine its estimation error. To achieve these goals, we have performed tests with a group including healthy volunteers and people with different degrees of disability. We have determined how they bear their weight on a rollator to check whether the proposed methodology is valid or not depending on their condition. In addition, we have performed tests using three different methods to estimate gait events simultaneously: an instrumented treadmill, an optical active-marker system and a rollator equipped with force sensors and encoders. Errors in the reference systems are defined, so we have compared their results with the rollator system to obtain the estimation error in the method proposed in [22].

## 2. Experimental Section

### 2.1. Volunteer Selection

**Figure 1:**
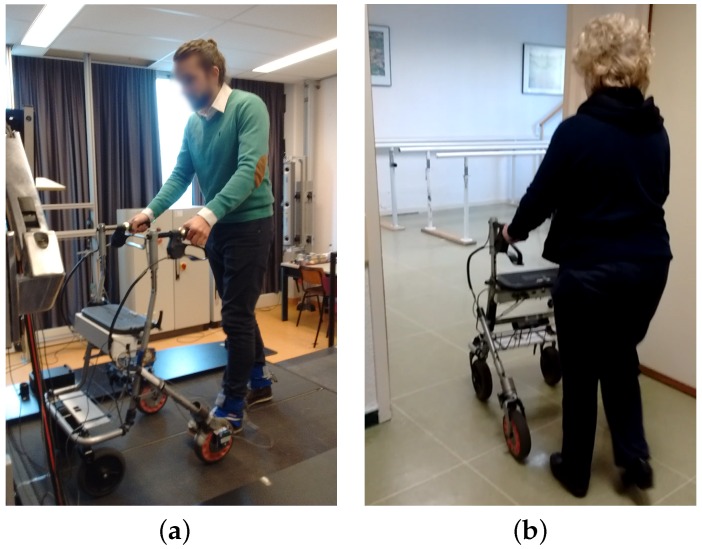
Healthy volunteer over a treadmill with two markers attached in the heel at Vrije Universiteit Amsterdam (VUA) (**a**); Amputee below knee using the i-Walker platform at Hospital Regional Universitario (HRU) (**b**).

This study consisted of two stages. In stage 1, volunteers were allowed to move freely using a smart rollator for support (Figure 1a). These tests included both healthy and challenged volunteers. Healthy volunteers were asked to emulate a challenged person’s behavior by increasing weight bearing on the rollator and by reducing gait speed, as proposed in [27]. Our challenged volunteers had to meet the following criteria: (i) they needed to be able to walk with a rollator; (ii) they needed experience using rollators; and (iii) they had to present a mild to severe disability. These measurements were performed to determine the target population for the force based gait analysis method.

In stage 2, a group of volunteers was asked to walk with the smart rollator over an instrumented treadmill using a smart walker, wearing optical markers, to obtain the estimation error of the force based gait analysis method. These tests were more challenging and uncomfortable and had to be conducted at a specific facility, so the number of volunteers with disabilities measured in this stage was limited.

The full test group for stages 1 and 2 included 43 volunteers from three different institutions. Most challenged volunteers (11 males and 18 females) were either inpatients from Fondazione Santa Lucia (FSL) in Italy or rehabilitation patients from the Hospital Regional Universitario of Malaga (HRU) in Spain. These 29 volunteers were on average 61.04±15.44 years old (range 31–86 years). They all presented musculoskeletal or neural impairments. Musculoskeletal impairments included: lower limb amputation (×3), polytraumatism (×2), fractures (prosthetic femur, intertrochanteric hip, intertrochanteric fracture femur), total hip replacement, spinal fusion hip arthroplasty, and rotate left leg. Neural impairments included: tetraparesis (×2), vestibular disorders (×3), dementia (mild or severe, ×3), Parkinson’s disease (mild or severe, ×6), stroke (×2), ischemia and multiple sclerosis. Twelve healthy and two challenged volunteers were recruited by word of mouth at ’Vrije Universiteit Amsterdam’ (VUA). Healthy volunteers were five males and seven females, with an average age of 24.5±4.5 years (range 20–32). The two challenged volunteers presented severe disabilities and were males, 55 and 69 years old. One had suffered a stroke and the other one had a vestibular disorder. All 43 volunteers were included in stage 1. Only volunteers from VUA were included in stage 2.

All 43 volunteers were evaluated using the Tinetti scale [28] to separate them into healthy (Figure 1a), mild or severe condition groups (Figure 1b). Healthy volunteers were those who obtained a perfect score on the Tinetti scale, i.e., they had a good balance control and a healthy gait pattern. Challenged volunteers were separated into those with a score <25 (mild to severe disabilities) and those with a score between 25 and 28 (minor to mild disabilities). This separation was based on different levels of fall risk [29].

### 2.2. Capture System

**Figure 2:**
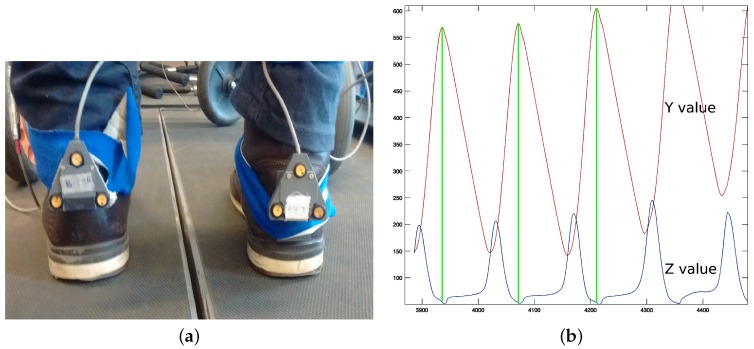

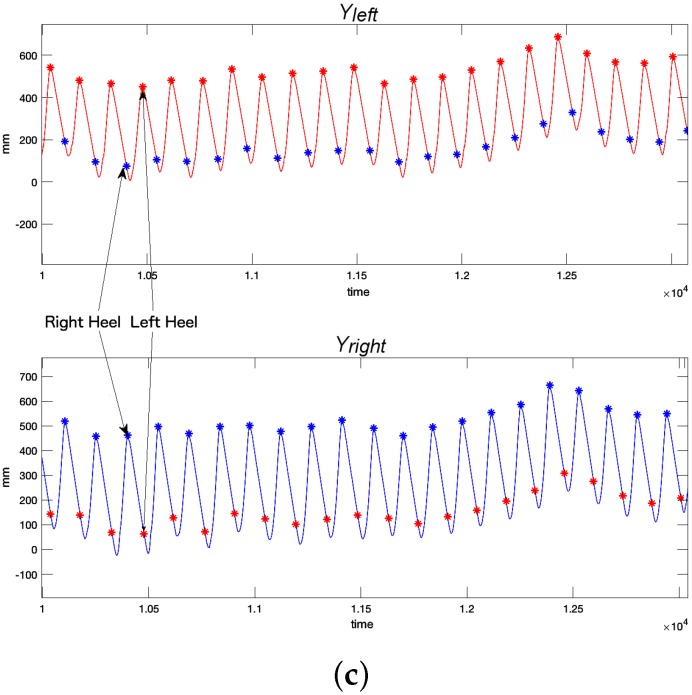
Optotrak gait analysis from a healthy volunteer. (**a**) Optotrak’s markers in the heels; (**b**) Optotrak’s Z and Y values of the left heel in user 6 and (**c**) top peaks in left ($Y_{left}$) and right ($Y_{right}$) functions.

**Figure 3:**
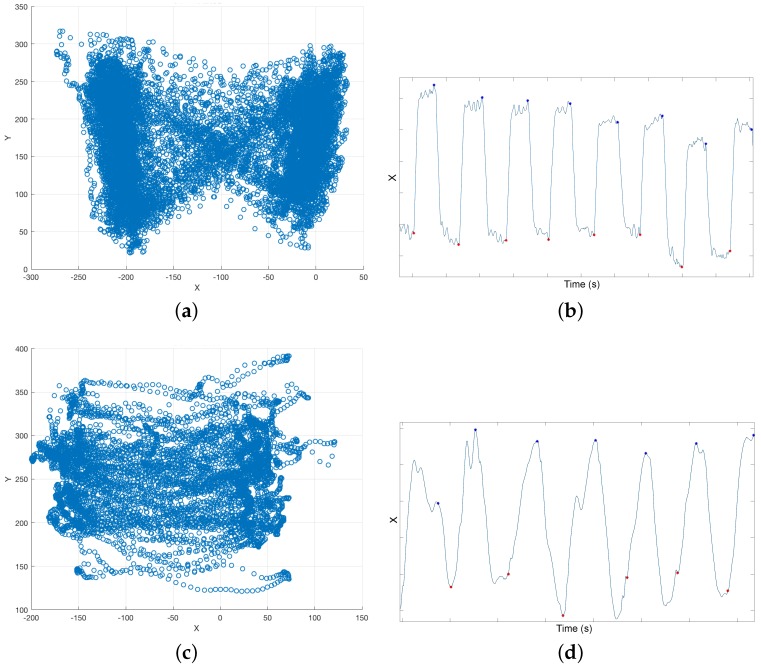
CoP and forces in a healthy volunteer who supports a similar effort in the handlebars during the test (**a**,**b**); CoP and forces in a challenged volunteer who does not support a similar weight on both handlebars during the test (**c**,**d**). (**a**) CoP in the rollator frame coordinate system; (**b**) X-axis in the CoP and detected peaks; (**c**) CoP in the rollator frame coordinate system; and (**d**) X-axis in the CoP and detected peaks.

In this study, three different systems were used for gait analysis:

1. i-Walker smart rollator: The i-Walker is a smart rollator developed at the *Universitat Politecnica de Catalunya* [30] based on a standard MEYRA^®^ (MEYRA GmbH, Kalletal, Germany) rollator frame. It includes a steel force transducer in the vertical stem of each handle to measure exerted forces on all three axes. Strain gauges connected in an additive full bridge are bonded to the transducer where stress is higher. The signal goes through a programmable gain instrumentation amplifier and is digitalised using a microcontroller. The system provides an accuracy of: 0.1 N (pushing); 0.2 N (transversal) and 0.05 N (resting force). In addition, each wheel has an encoder to estimate odometry. It includes an embedded system for data filtering and preprocessing that supports a wireless connection. All volunteers in both stages of our tests used the i-Walker rollator (Figure 1a,b).
2. Optotrak system: The Optotrak (NDI International, Waterloo, ON, Canada) is a system based on optical active-markers, which are recorded by external cameras. The markers coordinates in space were determined by the Optotrak software (NDI International, Waterloo, ON, Canada) with an accuracy below one millimeter and were sampled at 100 Hz. Volunteers from VUA walked on a treadmill with markers attached to their heels (Figure 1a). Figure 2a shows the active-markers attached to a volunteer’s heels. In addition, we attached a marker to each wheel of the rollator.
3. Force plates in the treadmill: Volunteers in stage 2 walked with the rollator on a treadmill equipped with force plates (Figure 1a), which provided a continuous trace of the center of pressure (CoP) trajectory. In our tests, the CoP is a combination of the volunteer’s and the rollator’s CoP. CoP traces are typically represented in a diagram known as the butterfly diagram (Figure 3a) [31]. Gait parameters were derived from the butterfly diagram.

### 2.3. Captured Spatiotemporal Gait Parameters

All three motion capture systems can return sets of spatiotemporal gait parameters depending on the employed methodology. The i-Walker is the most limited system because it relies on forces and odometry only. Hence, the i-Walker cannot return complex parameters like those related to body posture. All three capture systems, however, may detect the heel strike. Hence, we used heel strike as the basis to obtain the same spatiotemporal gait parameter set simultaneously from all three systems and, in doing so, assess our rollator based methodology.

Heel strike was determined differently with each capture system.

**Figure 4:**
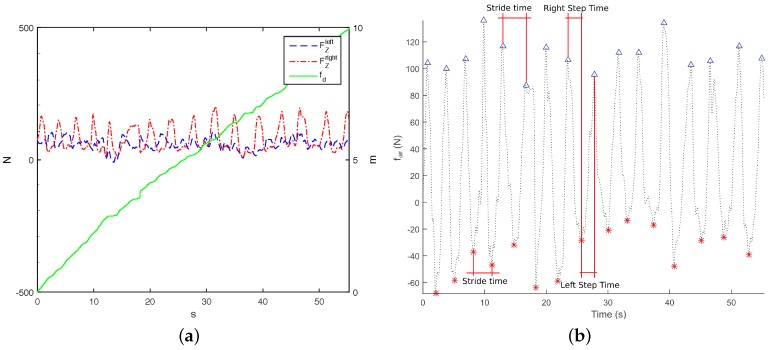
Spatiotemporal gait parameters on the i-Walker system in a user with polytraumatism in both lower limbs and psychological distress. (**a**) handlebar’s forces ($F_{Z}^{left}$, $F_{Z}^{right}$) and distance traveled $f_{d}$; and (**b**) the inflection points in $f_{diff}$ plotting over time are to be used for estimating the temporal gait parameters.

1. i-Walker: Force based heel strike detection in the i-Walker is based on the fact that when a person’s heel strikes, the handlebar force at the corresponding side grows, whereas the handlebar force in the opposite side decreases (Figure 4a). Hence, peaks in the forces difference function $f_{diff}=F_{Z}^{left}-F_{Z}^{right}$ correspond to heel strikesheel strikes. This function can be plotted against time but also against space if we consider odometry. Figure 4b shows how some temporal parameters can be obtained from these plots. This approach was successfully tested in [22].
2. Optotrak: Optotrak data can be used to estimate heel strike in a straightforward method. Volunteers had a marker on each heel (Figure 2a). When heel strike occurs, the Z (vertical) and Y (longitudinal) coordinates of the heel marker reach a local minimum or maximum (Figure 2b). In this work, we use the local maximum values of the $Y_{left,right}$ coordinates to detect heel strikes (Figure 2c).
3. Treadmill: When a user heel strikes on the treadmill, the CoP X (left–right) coordinate moves in the direction of the heel strike. Hence, heel strike can be detected as an inflection point in X coordinate time series (Figure 3c).

From heel strike data, we can obtain the following relevant parameters:

- Step time (SpT): time difference between a heel strike on one side and the next heel strike on the other side in seconds.
- Stride time (SdT): time difference between a heel strike on one side and the next heel strike on the same side in seconds.
- Number of Step (NoS): number of heel strikes.
- Time required (Tr): number of seconds that a volunteer takes to complete the test.
- Cadence (CAD): $60*\frac{NoS}{Tr}$.
- Step length (SpL): distance between a heel strike on one side and the next heel strike on the other side in meters.
- Stride length (SdL): distance between a heel strike on one side and the next heel strike on the same side in meters.
- Distance(*d*): sum of all step lengths.
- Average walking velocity (WV): $\frac{d}{Tr}$.

## 3. Results

### 3.1. Stage 1: Minimum Requirements for Force Based Gait Analysis

Many studies on assistive technologies are actually performed on healthy volunteers [5,19,27]. There are several reasons to justify this choice. Tests with challenged volunteers may be limited by ethical or practical considerations. Volunteers with neurological diseases or cognitive disorders, who cannot follow the test instructions, and volunteers with physical disabilities, who cannot walk for a long period of time, are often discarded. Tests that are deemed stressful or uncomfortable for volunteers—e.g., tiring tests, heavy wearables, active assistive devices, etc.—are often disapproved. Additionally, it is not always possible to bring people to lab facilities.

Unfortunately, healthy people present a very different walking behavior than persons with disabilities. Differences in gait between healthy and challenged volunteers may affect forces on the rollator frame and, hence, $f_{diff}$ values. If $f_{diff}$ is not well defined, heel detection and, consequently, force based gait analysis is not possible. The first stage of our experiments focuses on evaluating whether there are significant differences in terms of $f_{diff}$ depending on the user’s condition.

**Figure 5:**
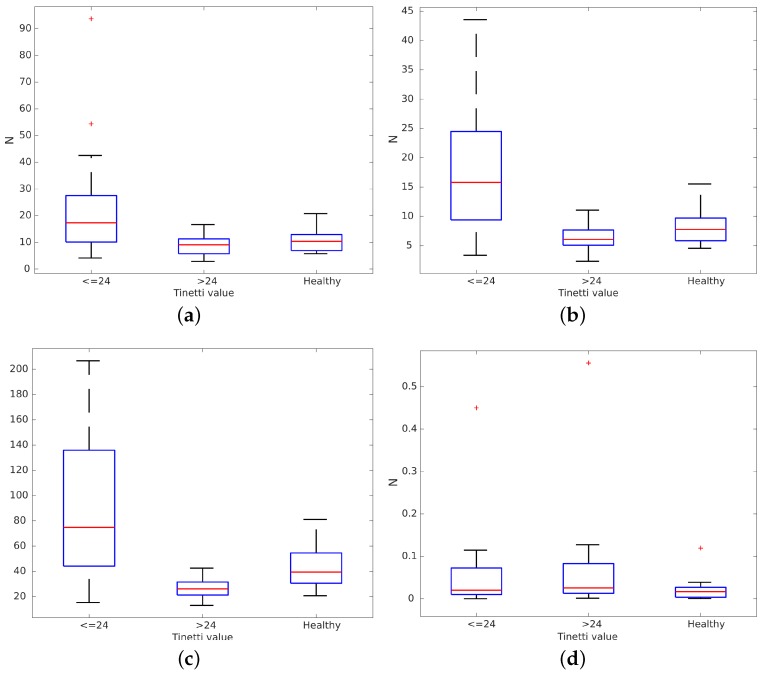
The differences in forces by volunteer $f_{diff}=F_{Z}^{left}-F_{Z}^{right}$ clustered by Tinetti score. (**a**) Average $f_{diff}$; (**b**) Variation $f_{diff}$; (**c**) Maximum $f_{diff}$ and; (**d**) Minimum $f_{diff.}$

Volunteers in FSL and HRU were asked to walk freely for three minutes in their rehabilitation rooms. Other patients and therapists were allowed to walk around these rooms at the same time. We recorded all data gathered from the rollator sensors during these tests. Paths in these tests were decomposed into straight lines to obtain behaviors like those from the volunteers who walked on the treadmill at VUA. Data from volunteers participating in the measurements on the treadmill were pooled with the data on straight line gait during over ground walking. Our 43 volunteers (31 challenged and 12 healthy ones) were split into three groups depending on their condition, as detailed in a further subsection: healthy, mild and severe disabilities. Then, we evaluated the average, variance, maximum and minimum of $f_{diff}$ for all three groups. Results are presented in Figure 5. There were differences between groups: ${\bar{f}}_{diff}$, $f_{diff}$ variance and maximum $f_{diff}$ were all higher for people with significant disabilities (Tinetti scores under 24). Differences in minimum $f_{diff}$ (Figure 5d) were not found because they were below the capture error in the handlebar force sensors (0.98 N) [22]. It can be observed that healthy volunteers tended to use the rollator like people with mild disabilities. However, they bore more weight on the rollator when they tried to emulate a non-healthy gait. It can be observed that their behavior is quite different from the group with Tinetti scores under 24, especially in terms of $f_{diff}$ variance and maximum.

We checked the normality of the data using the Lilliefors test. After that, we performed the F-test for equality of variances for each pair of groups (Severe-Healthy, Severe-Mild, Mild-Healthy) to test the null hypothesis that the two samples had the same variance. We found inequality of variance in some comparisons. Hence, we also performed a non-parametric two-sided Wilcoxon rank sum test for each pair of groups to test observed differences in their averages. Table 1 shows the *p*-value for the normality of the data per groups and for each comparison of $f_{diff}$ measurements with a significance level of α=0.05. The normality of the data has been validated for each group with a minimum *p*-value of 0.0587. The Lilliefors test could not reject the null hypothesis i.e., data coming from a normally distributed population. Hence, the F-test can be applied. Volunteers in the severe group clearly presented differences with respect to healthy volunteers both in $f_{diff}$ average and variation. The hypothesis of equal variance was also rejected for these variables. On the other hand, the hypothesis that mildly challenged volunteers have a similar median compared to healthy volunteers was rejected only for maximum $f_{diff}$. The same occurred for the variance hypothesis. Finally, the hypothesis that severely challenged volunteers have a similar median compared to the mildly challenged volunteers was rejected in all cases. In addition, the hypothesis of equality of variances was rejected for the variation and the maximum of $f_{diff}$.

Volunteers in the severe group clearly presented differences with respect to healthy volunteers both in averages and variation. The hypothesis of equal variance was also rejected for these variables. On the other hand, the hypothesis that mildly challenged volunteers have a similar median compared to healthy volunteers was rejected only for the maximum. The same occurred for the variance hypothesis. Finally, the hypothesis that severely challenged volunteers have a similar median compared to the mildly challenged volunteers was rejected in all cases. In addition, the hypothesis of equality of variances was rejected for the variation and the maximum of $f_{diff}$.

These results show that there are substantial differences in $f_{diff}$ between the severely challenged group on one hand and the healthy or mildly challenged groups on the other hand. Results also show how our healthy volunteers, which were trying to emulate challenged volunteers, are closer to volunteers presenting mild disabilities than to severely challenged ones, even though they bear more weight on the rollator than the second group. This confirms that people use a rollator very differently depending on their condition and also outline that force based gait analysis methods may not be appropriate for healthy people, nor for people with minor disabilities because weight bearing on the rollator does not vary enough between sides to reliably estimate heel strike. Experiments in stage 2 will determine how variable weight bearing must be for reliable force based gait analysis and what estimation error can be expected in these cases.

### 3.2. Error Analysis

In stage 2, volunteers were asked to walk on a treadmill equipped with force plates using the i-Walker for support while they were tracked by the Optotrak system. These tests allowed us to further elaborate on the differences between healthy and challenged user groups.

All three systems were synchronized to extract the gait parameters previously described simultaneously. Results from the reference systems were used to evaluate the error in the rollator based estimation. The error function for each spatiotemporal parameter was equal to the difference between the value obtained from the rollator and the one obtained from one or the other reference system at each sample. The error function was determined for each volunteer and each spatiotemporal gait parameter separately. Subsequently, the error for the whole set of volunteers for a given spatiotemporal gait parameter *ρ* was characterized by its average $\varepsilon_{av}^{\rho }$ and its variance $\varepsilon_{sd}^{\rho }$. During averaging, positives and negatives errors cancel each other, so $\varepsilon_{av}^{\rho }$ is reduced. However, the combination of all errors in the capture process tends to make $\varepsilon_{sd}^{\rho }$ larger.

The Optotrak system measured at 100 Hz with a spatial error under one millimeter. To take the Optotrak error into account, $\varepsilon^{\rho }$ will be increased by 0.001 m for the spatial parameters and by 0.01 s for the temporal parameters.

**Figure 6:**
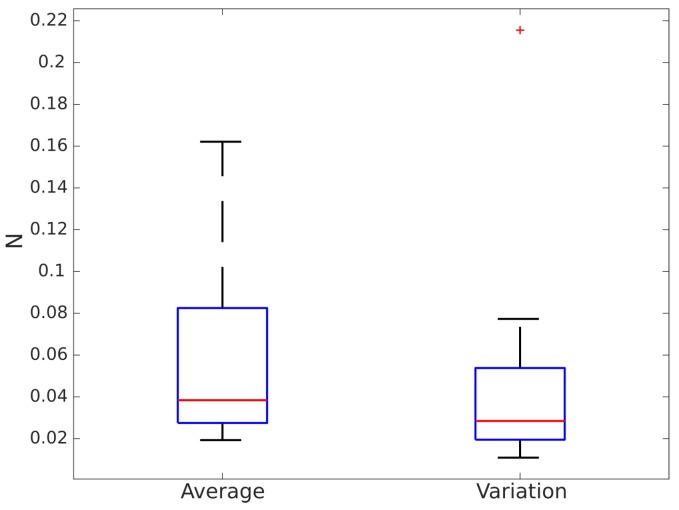
Heel strike differences between the treadmill and the Optotrak system.

The treadmill had a higher sampling rate than the Optotrak: it measured the CoP, and, consequently, heel strike, at 200 Hz. However, in our experiments, we found that the CoP returned by the treadmill was not always accurate. Figure 6 shows heel strike time differences between the treadmill and the Optotrak for heel detection. These differences are clearly above the error in the Optotrak system (0.01 s). Heel strike could be obtained accurately from the CoP only for some healthy users. If volunteers did not bear weight on the rollator, their CoP had the traditional butterfly shape (Figure 3a). If healthy users increased weight support on the rollator to emulate a challenged user’s behavior, the plot became a butterfly with an offset. Healthy users tended to support exactly the same weight on both handlebars when they walked. Challenged volunteers, on the contrary, bore more weight on one handlebar or the other while they walked. In these cases, the CoP could not be modelled as a butterfly shape, and, hence, the CoP based heel detection was inaccurate (Figure 3d). In brief, treadmill based estimation was accurate in cases where the rollator based estimation is likely not reliable and vice versa. Hence, errors were evaluated using only the differences between the Optotrak and the i-Walker derived parameters.

Table 2 shows the average and variance of the relative errors for each obtained gait parameter per volunteer using the Optotrak system as a reference. Variance was very high with respect to the average, especially for healthy volunteers. The table also shows how errors were substantially larger for some (usually healthy) volunteers (9—Healthy, 10—Healthy) when compared to others (12—Healthy, 1—Challenged). This may be due to them forgetting to emulate a challenged person’s behavior at some points, and, hence, not bearing enough weight on the rollator. In these cases, any force based methodology would become inaccurate.

**Figure 7:**
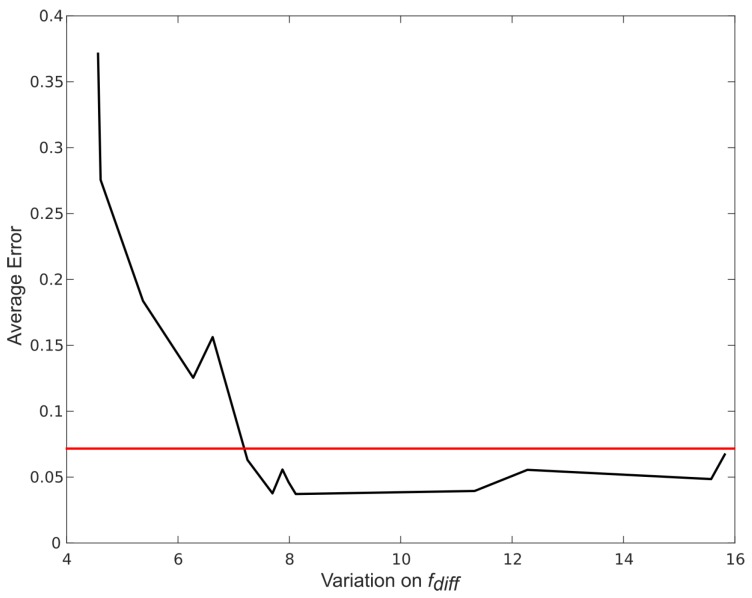
The average relative error of gait parameters obtained from the i-Walker order of increasing variation on $f_{diff}$.

**Figure 8:**
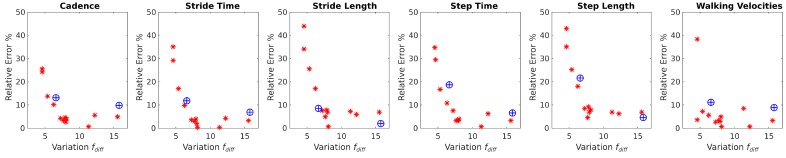
Relative error of the i-Walker parameters compared with the Optotrak system in challenged volunteers (⊕) and healthy volunteers (*).

As described previously, our force based gait analysis algorithm searches for maximum differences between handlebar forces to estimate spatiotemporal heel strikes. When volunteers bear more weight on the rollator, the variation of these forces is higher and heel strike detection is more accurate. Figure 7 and Figure 8 show how the estimation error in spatiotemporal gait parameters averaged over all parameters and for each specific parameter decrease when the variance in the difference between handlebar’s forces ($f_{diff}$) increases. The average relative error in the spatiotemporal gait parameters tended to stabilize when $f_{diff}$ was above 7 N (Figure 7). The maximum relative error values for each gait parameter (Figure 8) when $f_{diff}$ was above 7 N were:

- Cadence: 9.14%;
- Stride time: 7.04%;
- Stride length: 7.88%;
- Step time: 7.65%;
- Step length: 9.11%;
- Walking Velocities: 8.8%.

## 4. Discussion

After relative error in gait parameter estimation is analyzed using all three different capture systems, one can immediately find major differences between healthy users and challenged subjects using these systems. In most cases, challenged users bear more weight on the rollator, even when healthy users are purposefully trying to do so. In addition, challenged users bear weight asymmetrically on the handlebars, i.e., $f_{diff}$ variance is much larger, whereas healthy users tend to be symmetrical. Consequently, the treadmill is fit to detect heel strike for healthy people, but not for challenged users with mild to severe asymmetries. Hence, it is advisable to use only Optotrack for benchmarking.

**Figure 9:**
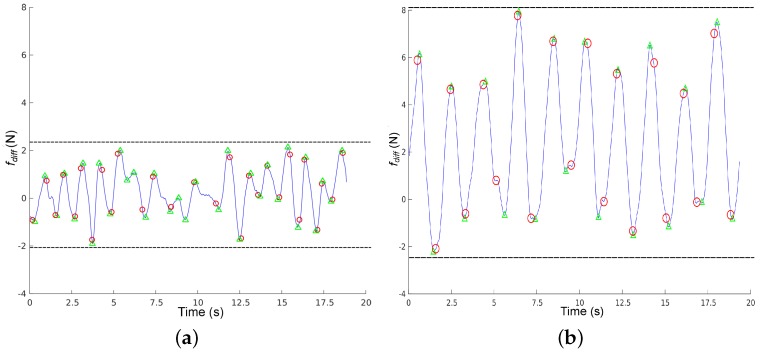
Volunteer heel strikes plotted over $f_{diff}$. The detection of heel strikes are plotted as: △ for the i-Walker and ◯ for Optotrak system. (**a**) Healthy volunteer and; (**b**) challenged volunteer with a stroke. Left side support more weigth.

Figure 9 shows 20 s of data captured from a healthy volunteer and a challenged one (stroke). We can observe that $f_{diff}$ for the healthy volunteer is small and centred around 0, meaning that force variance between the handlebars is reduced. Under these circumstances, detection is not reliable: we can observe that the force-based method has detected four erroneous steps around time instant 10, where the function is smallest. $f_{diff}$ for the challenged volunteer has a larger amplitude (slightly over 7 N), and we can observe that it is centered around 2, meaning that this subject bears more weight on the left side of the rollator. In this case, steps are correctly detected by the force based method with respect to the Optotrack. We can also observe that $f_{diff}$ is more regular for the challenged user than for the healthy one. This was to be expected, since healthy volunteers do not really need support and they have to keep in mind that they need to bear some weight on the rollator. Indeed, during these 20 s, the weight variance in the handlebars for the healthy volunteer was less than 0.5 Kg on average, whereas it was around 1 Kg for the challenged volunteers most of the time, just within the 7 N threshold required for reliable force based step detection.

Results from stage 2 suggest that, as long as the difference between the weight that people bear on the rollator’s handlebars is large enough, all gait parameters can be obtained using the methodology proposed in [22] using only the i-Walker with less than a 10% error with respect to the Optotrak system. In stage 1, the restriction of a variation above 7 N was satisfied by: 95.83% of challenged volunteers with a Tinetti score below 24, 37.5% of challenged volunteers with a Tinetti score above 24% and 66.67% of healthy volunteers feigning a challenged gait. It can be concluded that rollator based gait analysis is reliable for people presenting mild to severe disabilities and also that, as long as results for people presenting $f_{diff}$ lower than 7 N are discarded, estimation errors remain below 10%.

It is interesting to note that when healthy people are feigning a challenged behavior, they might bear more weight on the rollator than challenged volunteers. However, the variance of the force difference on the handlebars is reduced, meaning that they are using the rollator like a trolley rather than as a support device. Actually, $f_{diff}$ for healthy users is more similar to people with minor disabilities than to volunteers presenting mild to severe disabilities, despite how much weight they are bearing on the device.

In general, tests with healthy volunteers to validate assistive devices are not recommended because systematic differences in sensor readings may cause bias. However, if it is impossible to work with challenged volunteers, at the very least, it is necessary to compare the system input instance for healthy volunteers with reported inputs for mildly to severely challenged volunteers. If instances are similar, it can be concluded that healthy volunteers are feigning challenged behavior acceptably for the test.

There are alternative methods to obtain gait parameters in rollators using using onboard RGB-D cameras instead of force sensors [32]. However, reported average error ranges from 0.1 s to 0.8 s in temporal parameters and from 0.02 m to 0.04 m in spatial parameters. If we use the Optotrak gait parameters as a reference, given the Optotrak spatial gait parameters $p_{s}$, relative error would be bounded by $e_{min}^{s}=\frac{p+0.02}{p}$ and $e_{max}^{s}=\frac{p+0.04}{p}$. In addition, given the Optotrak temporal gait parameters $p_{t}$, relative error would be bounded by $e_{min}^{t}=\frac{p+0.1}{p}$ and $e_{max}^{t}=\frac{p+0.8}{p}$. Table 3 shows the relative errors compared to our method. It can be observed that both methods present similar relative errors for minimum values of the parameters. However, there are major differences in relative error for maximum values of temporal gait parameters. These results are coherent with the conclusions in [32].

## 5. Conclusions

In this study, we analyzed the validity of a force based methodology for gait analysis using a smart rollator. The aims of this study were to determine whether this methodology can be applied to any types of users regardless of their condition and to determine the estimation error. In the first stage, we studied the differences in weight bearing on rollator handlebars between 12 healthy volunteers and 31 challenged volunteers presenting a variety of musculoskeletal and/or neurological disabilities. Despite obvious gait differences, healthy volunteers are often used in studies on assistive devices. In some studies, they are asked to emulate a challenged user’s behavior by bearing more weight on the devices and walking slower. However, our study shows that, even in this case, there are major differences in terms of how people bear weight on a rollator depending on their condition.

In a second stage, we focused on determining how users need to bear weight on the walker for accurate gait analysis and which estimation errors appear in these cases. To achieve this goal, we extracted the same spatiotemporal gait parameters from heel strikes detected simultaneously using: (i) the i-Walker smart rollator; (ii) an (optical) Optotrak system; and (iii) a treadmill equipped with force plates. These tests were done on a subset of 12 healthy volunteers and two challenged volunteers. These volunteers walked on the treadmill wearing Optotrak markers on their heels and using the i-Walker.

First, we found out that heel strike on the treadmill could not be reliably detected for challenged users because they do not bear weight symmetrically on both handlebars. Hence, estimation errors were obtained by comparing gait parameters extracted from the rollator with those extracted from the Optotrak system, which reportedly presents a spatial error lower than 1 mm.

Our conclusions support the idea that force based gait analysis on a rollator is accurate (less than 10% error) as long as peak force differences between the handlebars are over 7 N. This condition was satisfied by 95.83% of our volunteers presenting mild to severe disabilities (Tinetti scores under 24). Only 37.5% of challenged volunteers with a Tinetti score above 24, mostly including people at the end of their rehabilitation period, satisfied this restriction. Thus, force based gait analysis using a rollator would not be advisable for this group. Finally, 66.67% of our healthy volunteers met this restriction. In general, tests with healthy volunteers to validate assistive devices are not recommended because systematic differences in the sensor readings may cause bias.

## Figures and Tables

**Table 1:** Lilliefors test/Mann–Whitney U (M.W.U.) test/F-Test *p*-values. The null hypotheses rejection are been marked.

Test	Groups		$F_{\mathrm{diff}}$		
			Average	Variation	Maximum
Lillie. T	Healthy	-	0.0949	0.0587	0.0882
	Mild	-	0.47000	0.4190	0.4913
	Severe	-	0.3607	0.4344	0.4179
M.W.U	Severe	Healthy	0.0276	0.0155	0.1086
	Mild	Healthy	0.4179	0.1535	0.0228
	Severe	Mild	0.0225	0.0027	0.0013
F-test	Severe	Healthy	0.0479	0.0165	0.2646
	Mild	Healthy	0.7996	0.5208	0.0465
	Severe	Mild	0.0618	0.0124	0.0061

**Table 2:** Relative error for each gait parameter obtained from the i-Walker with respect to the Optotrak per volunteer.

Volunteer	CAD	*SdT*		*SdL*		*SpT*		*SpL*		*WV*
		*Avg*	*Var*	*Avg*	*Var*	*Avg*	*Var*	*Avg*	*Var*	
1Healthy	0.0551	0.0429	3.9747	0.0577	1.9367	0.0619	6.5305	0.0634	1.7652	0.0080
2Healthy	0.0383	0.0022	2.6964	0.0079	0.8769	0.0409	4.0643	0.0801	1.6157	0.0068
3Healthy	0.0327	0.0308	2.8290	0.0479	0.2552	0.0332	3.5084	0.0452	0.4980	0.0332
4Healthy	0.0432	0.0375	2.0081	0.0769	0.2996	0.0765	2.4314	0.0843	0.6561	0.0248
5Healthy	0.0461	0.0381	4.3374	0.0788	1.2538	0.0351	5.7775	0.0911	2.2610	0.0306
6Healthy	0.1379	0.1692	10.6766	0.2555	4.8696	0.1658	11.0865	0.2516	5.6298	0.0722
7Healthy	0.0061	0.0028	1.4610	0.0723	0.2589	0.0056	2.4345	0.0694	0.8294	0.0846
8Healthy	0.1010	0.0998	14.1404	0.1714	3.9903	0.1068	11.7322	0.1806	3.8392	0.0562
9Healthy	0.2545	0.3521	20.5426	0.4383	4.3025	0.3460	18.7731	0.4289	5.1066	0.3827
10Healthy	0.2416	0.2902	12.8386	0.3420	7.7650	0.2963	15.9136	0.3502	9.7728	0.0372
11Healthy	0.0252	0.0197	2.1781	0.0704	0.3320	0.0344	3.7858	0.0675	0.9522	0.0503
12Healthy	0.0497	0.0325	5.6370	0.0683	1.3788	0.0340	5.8574	0.0690	1.7221	0.0314
1Challenged	0.0914	0.0704	1.0939	0.0210	0.4393	0.0644	2.0162	0.0469	0.1719	0.0880
2Challenged	0.1301	0.1181	0.1333	0.0839	0.2055	0.1878	0.2629	0.2151	0.3269	0.1122

^1^ Cadence; ^2^ Stride Time; ^3^ Stride Length; ^4^ Step Time; ^5^ Step Length; ^6^ Walking Velocity; ^a^ Average; ^b^ Variance.

**Table 3:** Estimation of a relative error using an RGB-D camera and the Optotrak gait parameters as a reference.

Gait Parameters	Minimum Error	Maximum Error	Force Sensors Error
Stride Time	6.84%	54.69%	7.04%
Stride Length	4.77%	9.54%	7.88%
Step Time	13.77%	110.12%	7.65%
Step Length	9.54%	19.09%	9.11%
